# Osmostress-Induced Apoptosis in *Xenopus* Oocytes: Role of Stress Protein Kinases, Calpains and Smac/DIABLO

**DOI:** 10.1371/journal.pone.0124482

**Published:** 2015-04-13

**Authors:** Nabil Ben Messaoud, Jicheng Yue, Daniel Valent, Ilina Katzarova, José M. López

**Affiliations:** Institut de Neurociències, Departament de Bioquímica i Biologia Molecular, Unitat de Bioquímica, Facultad de Medicina, Universitat Autònoma de Barcelona, 08193 Cerdanyola del Vallès, Barcelona, Spain; The University of Texas MD Anderson Cancer Center, UNITED STATES

## Abstract

Hyperosmotic shock induces cytochrome c release and capase-3 activation in *Xenopus* oocytes, but the regulators and signaling pathways involved are not well characterized. Here we show that hyperosmotic shock induces rapid calpain activation and high levels of Smac/DIABLO release from the mitochondria before significant amounts of cytochrome c are released to promote caspase-3 activation. Calpain inhibitors or EGTA microinjection delays osmostress-induced apoptosis, and blockage of Smac/DIABLO with antibodies markedly reduces cytochrome c release and caspase-3 activation. Hyperosmotic shock also activates the p38 and JNK signaling pathways very quickly. Simultaneous inhibition of both p38 and JNK pathways reduces osmostress-induced apoptosis, while sustained activation of these kinases accelerates the release of cytochrome c and caspase-3 activation. Therefore, at least four different pathways early induced by osmostress converge on the mitochondria to trigger apoptosis. Deciphering the mechanisms of hyperosmotic shock-induced apoptosis gives insight for potential treatments of human diseases that are caused by perturbations in fluid osmolarity.

## Introduction

Cells have been submitted to osmotic stress from the very beginning of their formation, compromising their function. Hyperosmolarity has many damaging effects on cells by promoting water flux out of the cell, triggering cell shrinkage and intracellular dehydratation [1]. Therefore, it is expected that cells had developed several mechanisms to adapt osmotic changes for surviving [2]. However, when the osmotic shock is intense or persistent the cell machinery can engage a cell death program.

It is known that hyperosmolar stress triggers apoptosis in a wide variety of cells [3–7] and is involved in several human diseases: diabetes, inflammatory bowel disease, hypernatremia, and dry eye syndrome [2]. The studies concerning osmostress-induced apoptosis suggest a variety of mechanisms, depending on the cell type considered. However, it has not been defined how many mechanisms operate at the same time or in a progressive and coordinated manner in a particular cell type. There are no reports pointing how the integration of different pathways, activated by hyperosmotic shock, might converge on cell death.

We have reported that hyperosmotic stress induces cytochrome c release and caspase-3 activation in *Xenopus laevis* oocytes [8]. Important players that may regulate cell death, and whose main features are presented here, are stress protein kinases, calpains, Smac/DIABLO, and cytochrome c.

The c-Jun NH_2_-terminal kinases (JNKs) and the p38 mitogen-activated protein kinases (p38 MAPKs) are a group of the family MAP kinases activated by dual phosphorylation of a tripeptide motif Thr-Pro-Tyr (JNK) or Thr-Gly-Tyr (p38) by different MKKs, which in turn are activated by several MAPKKKs (for example, MEKK1) [9]. JNK and p38 can have a pro- or an anti-apoptotic function depending upon the stimuli and the cellular context [10,11]. It has been shown that early transient activation of JNK or p38 promotes cell survival, whereas prolonged activation can mediate apoptosis [12–14]. Although JNK and p38 are activated during hyperosmotic shock in almost all cell types, their role in osmostress-induced apoptosis is not clear.

Calpains are Ca^2+^-activated non-lysosomal cysteine proteases that participate in a variety of cellular processes including remodeling of cytoskeletal/membrane attachments, different signal transduction pathways and apoptosis [15,16]. Interestingly, hyperosmotic shock induces a rapid and transient increase of Ca^2+^ in the cytosol of several mammalian cell types [17–19]. However, it is not clear whether calpain activation is a general feature of hyperosmotic shock and how relevant it can be in osmostress-induced apoptosis.

Smac/DIABLO is a mitochondrial protein located in the intermembrane space, and under stress conditions is released into the cytosol and binds to various inhibitor of apoptosis proteins (IAPs), neutralizing their inhibitory effect on caspases and triggering cell death [20,21]. Cytochrome c is present as loosely and tightly bound pools attached to the inner mitochondrial membrane by its association with cardiolipin [22,23]. In cells submitted to stress, cytochrome c is also released from mitochondria and facilitates the apoptosome formation and subsequent capase-3 activation. However, the kinetics of release of cytochrome c and Smac/DIABLO shows high variation, depending on the study. It has been reported that citotoxic drugs and UVB-irradiation induce cytochrome c release before Smac/DIABLO, whose efflux from mitochondria would require active caspases [24,25]. It is also reported simultaneous release of both proteins in response to different stimuli in MCF-7 and HeLa cells [26–28], or early Smac/DIABLO release in response to cephalostatin [29]. To our knowledge, there are no studies comparing the kinetics of Smac/DIABLO and cytochrome c release induced by hyperosmotic shock.

In the present work, we analyze in detail the time-course events during osmostress-induced apoptosis in *Xenopus* oocytes and the role of stress protein kinases, calpains, and Smac/DIABLO.

## Materials and Methods

### Oocyte isolation and treatment

Oocytes were obtained from sexually mature *Xenopus laevis* females (purchased from Centre d’Elevage de Xenopes, Montpellier, or from Xenopus Express, Vernassal, France), anesthetized in 0.02% benzocaine and portions of ovary were removed through a small incision on the abdomen. The incision was sutured and the animal was returned to a separate tank until it had fully recovered from the anaesthesia. It was then returned to a large tank in which all the frogs were kept for at least 4 weeks until the next surgery. The protocol was approved by the Committee on the Ethics of Animal Experiments of the Universitat Autònoma de Barcelona (Permit Number: CEEAH 439) and all efforts were made to minimize animal suffering. The tissue was examined to ensure the ovaries were healthy and dissected in small pieces. Oocytes were defolliculated for 2–3 h at room temperature with collagenase/dispase (0.8 mg/ml (Sigma), 0.48 mg/ml (Roche)) in MBS (5 mM HEPES, 88 mM NaCl, 1 mM KCl, 1 mM MgSO_4_·7H2O, 2.5 mM NaHCO_3_, 0.7 mM CaCl_2_, pH 7.8) with agitation. The oocytes were then washed thoroughly with MBS and transferred to petri dishes. Stage VI oocytes were sorted manually and incubated overnight in MBS at 18°C. The next day, healthy survivors were selected and transferred to a petri dish containing fresh MBS. Oocytes were exposed to hyperosmotic shock by transferring them to a new dish containing MBS with 300 mM sorbitol, collected at different times, and treated as described below. Some oocytes were incubated with drugs dissolved in MBS at the concentrations and times indicated or injected with cRNAs (capped RNAs) and exposed to hyperosmotic shock.

### Inhibitors

SB203580, SP600125, ALLN, Z-DEVD.fmk (all from Calbiochem), BIRB796 (Axon Medchem), MDL28170 (Sigma), and Z-VAD.fmk (Bachem) were dissolved in DMSO to prepare stock solutions. Oocytes were pre-incubated for 1 h with the corresponding inhibitor dissolved in MBS to a final concentration of 100 μM, and then incubated for the indicated times with the same concentration of inhibitor dissolved in sorbitol (300 mM). We used a higher concentration of inhibitors compared to mammalian cells due to specific properties of *Xenopus* oocytes (presence of vitelline membrane and the yolk) that reduce the actual concentration of drugs at the cell membrane. In general, IC_50_ values are approximately 10 to 20-fold higher when the drugs are applied to the extracellular surface of *Xenopus* oocytes [30,31].

### Oocyte lysis and Western blot analysis

Fresh oocytes were lysed by pippeting up and down in 200 μl (pools of 20 oocytes) of ice-cold extraction buffer (0.25 M sucrose, 0.1 M NaCl, 2.5 mM MgCl_2_, 20 mM HEPES, pH 7.2) containing 1 mM EDTA, 1 mM EGTA, protease inhibitors (10 μg/ml leupeptin, 1 mM PMSF, 10 μg/ml aprotinin) and phosphatase inhibitors (50 mM β-glycerolphosphate, 50 mM sodium fluoride, 1 mM sodium orthovanadate, 5 mM sodium pyrophosphate). Samples were clarified by centrifugation at 14.500 rpm for 5 min and supernatants were collected and processed for immunoblotting or caspase assay as described below. The whole supernatants were denatured with Sample Buffer (50 mM Tris HCl, pH 6.8, SDS 2%, 100 mM dithiothreitol, 10% glycerol) and subjected to 10% or 15% SDS/PAGE and transferred to Immobilon-P membranes (Millipore). Native polyacrylamide gels (3% upper gel, 10% lower gel) were prepared by dissolving acrylamide/bis-acrylamide in Tris HCl pH 8.8 (0.375 M final concentration). Samples were diluted 1:1 with Sample Buffer 2X (62.5 mM Tris HCl, pH 6.8, 25% glycerol, 1% bromophenol blue), spin down to avoid precipitates, and loaded in the gels. Electrophoresis for native gels was performed in 1X Running Buffer (25 mM Tris, 192 mM glycine, pH 8.3) at 175 V, and transfer of proteins to Immobilon-P membranes was performed with the usual SDS transfer buffer. Uniformity of samples loading was verified by Ponceau (Sigma) staining of the blots. Membranes were blocked for 1 h with 5% dried skimmed milk in TBST (50 mM Tris, 150 mM NaCl, 100 mM KCl, pH 7.4, and 0.1% Tween 20) and then incubated with the following polyclonal antibodies from Cell Signaling: anti-AMPKα (2532), anti-pAMPKα (Thr172) (2531), anti-pp38 (Thr180/Tyr182) (9211), anti-pJNK (Thr183/Tyr185) (9251), anti-JNK (9252), and anti-cleaved caspase-3 (Asp175) (9661). Polyclonal anti-p38 (sc-7149, Santa Cruz), polyclonal anti-Smac/DIABLO (2409, ProSci), monoclonal anti-Myc (M4439, clone 9E10, Sigma), monoclonal anti-β-actin (A19789, Sigma), monoclonal anti-fodrin α (Q13813, Millipore), monoclonal anti-ATP-synthase α (A21350, Invitrogen), and monoclonal anti-cytochrome c (556432, BD Pharmingen) were also used. Antibody binding was detected with horseradish peroxidase–coupled secondary antibody and the enhanced chemiluminescence (ECL) detection kit (Amersham).

### Mitochondrial and cytosolic fractions

For subcellular fractionation, 30 oocytes were lysed in 300 μl of ice-cold extraction buffer, as previously described, and samples were centrifuged at 1.000 g for 10 min at 4°C to remove lipids and the yolk. The supernatant was isolated and centrifuged at 16.000 g for 30 min at 4°C. The supernatant obtained (cytosolic fraction) was stored at -20°C, and the pellet (mitochondrial fraction) was resuspended in 50 ul of lysis buffer and stored at -20°C.

### Assay for DEVDase activity

Caspase-3 activity was measured in terms of assayed DEVDase activity using the synthetic peptide Ac-DEVD-AMC from Peptide Institute. Briefly, 25 μl of each cytosolic fraction (corresponding to 2.5 oocytes) were diluted to a final volume of 100 μl with a buffer containing 0.1 mM Ac-DEVD-AMC, 5% glycerol, 1 mM DTT, 10 mM HEPES pH 7.5 (final concentration) and incubated for 1h at 37°C. Fluorescence was measured at 360 nm for excitation and at 460 nm for emission.

### DNA constructs and in vitro transcription

Human MKK6-DD and MKK6-DA cloned in Ftx4 plasmid have been described previously [32]. MKK6-DD is a constitutively active kinase with the two phosphorylation sites in the activation loop Ser-207 and Thr-211 changed to Glu, whereas MKK6-DA is catalytically inactive by mutation of Glu-197 to Ala. Constitutively active mouse MEKK1 (MEKK1+) and catalytically inactive (MEKK1-KM) have been reported [33]. MEKK1+ was obtained by deletion of aminoacids 1 to 351 from wild type MEKK1, whereas catalytically inactive MEKK1 (MEKK1-KM) was obtained from MEKK1+ by mutation of Lys-432 to Met. Both MEKK1 DNAs were cloned into Ftx5 plasmid, which contains a myc tag in the N-terminus. In vitro transcriptions of capped RNAs (cRNAs) were performed by using mMessage mMachine T7 Transcription Kit (Ambion).

### Oocyte injection

Stage VI *Xenopus* oocytes were microinjected near their equator with 50 nl (5 ng) of the corresponding cRNAs. Injected oocytes were incubated for 18 h at 18°C and pools of 20 oocytes were collected before and after osmotic shock treatment (300mM sorbitol) for the indicated times. Oocytes were lysed and analyzed by Western blot and caspase-3 activity, as previously described. In some experiments oocytes were injected with antibodies anti-Smac/DIABLO (2409, ProSci), anti-Smac/DIABLO (567365, Calbiochem), anti-human IgG (109-001-008, Jackson ImmunoResearch), or rabbit IgG (I5006, Sigma).

### Statistical analysis

Data are expressed as means ± SEM. Paired t-test was used to compare caspase-3 activity in oocytes treated with or without inhibitors. One-way ANOVA with a Dunnett Multiple Comparison Test was used in oocytes microinjected with different cRNAs comparing all columns versus water injected oocytes. Values of p<0.05 were considered to be statistically significant.

## Results

### Hyperosmotic shock induces rapid release of Smac/DIABLO, calpain activation, p38 and JNK activation

**Fig 1:**
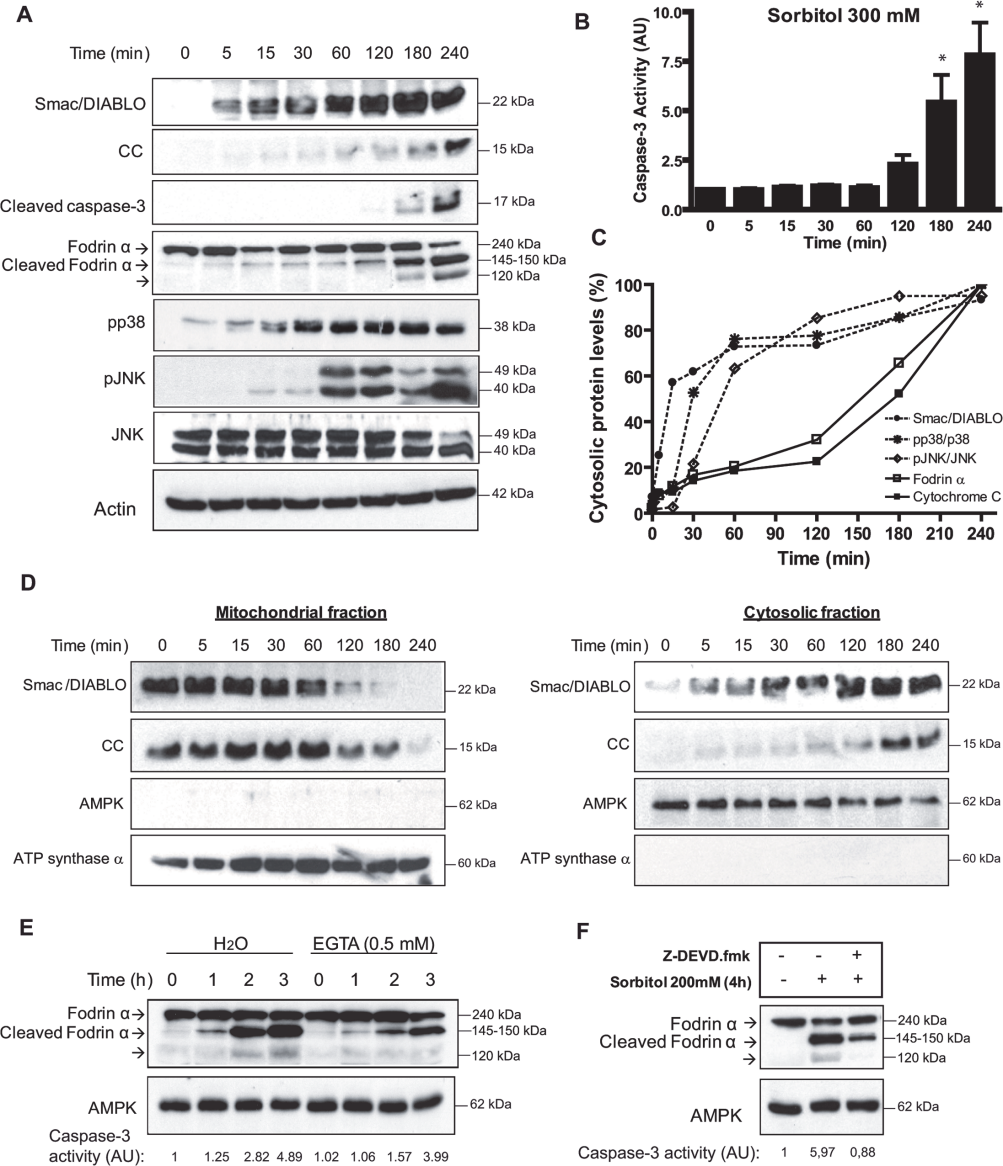
Hyperosmotic shock induces rapid release of Smac/DIABLO, calpain activation, p38 and JNK phosphorylation. A. Osmostress induces rapid release of Smac/DIABLO, calpain activation, p38 and JNK phosphorylation. Smac/DIABLO, cytochrome c (CC), cleaved caspase-3, fodrin α (240 kDa), cleaved fodrin α (145–150 kDa), pp38, pJNK, JNK, and actin (loading control) were determined by Western blot at different times in oocytes treated with 300 mM sorbitol. B. Time-course of caspase-3 activation induced by hyperosmotic shock. Oocytes were treated with 300 mM sorbitol and caspase-3 activity was determined giving value 1 to non treated oocytes. Data are represented as mean ± SEM, (n = 6), *p<0.05 (t-test) comparing with non treated oocytes. C. Kinetics of Smac/DIABLO and cytochrome c release, p38 and JNK phosphorylation, and fodrin α proteolysis induced by hyperosmotic shock. Oocytes were exposed to 300 mM sorbitol for 4 h and samples were collected at different times to analyze Smac/DIABLO and cytochrome c release, p38 and JNK phosphorylation, and fodrin α proteolysis (as a marker of calpain activation) by Western blot. Quantitative analysis was performed in the blots from three independent experiments and the average values represented as the percentage of protein levels for Smac/DIABLO, cytochrome c and fodrin α (145–150 kDa band), or as the ratio of pp38/p38 and pJNK/JNK, giving 100% to the highest value obtained in the time-course experiment. D. Hyperosmotic shock induces Smac/DIABLO release from the mitochondria. Oocytes were treated with 300 mM sorbitol, and cytosolic and mitochondrial fractions were obtained at different times as described in Materials and Methods. Smac/DIABLO and cytochrome c (CC) were analyzed by Western blot. AMPK and ATP synthase α were analyzed as markers of cytosolic and mitochondrial fractions, respectively. E. EGTA inhibitis calpain activation induced by osmostress. Oocytes were injected with EGTA (0.5 mM final concentration) or H_2_O (control) and 1 h later treated with 300 mM sorbitol and samples were collected at different times. Fodrin α (240 kDa), cleaved fodrin α (145–150 kDa), and AMPK (loading control) were determined by Western blot. Caspase-3 activity was determined as reported. F. Caspase-3 proteolyzes fodrin α. Oocytes were treated with 200 mM sorbitol for 4 h in the presence or absence of the caspase-3 inhibitor Z-DEVD.fmk (50 μM). Fodrin α (240 kDa), cleaved fodrin α (145–150 kDa), and AMPK (loading control) were determined by Western blot. Caspase-3 activity was determined as reported. Western blots in all figures are representative of at least three independent experiments.

We have reported previously that hyperosmotic shock induces cytochrome c release and caspase-3 activation in *Xenopus* oocytes between 2 and 4 h [8]. We analyzed in a time-course experiment the cytosolic levels of Smac/DIABLO and cytochrome c in *Xenopus* oocytes treated with 300 mM sorbitol. Hyperosmotic shock induced a significant increase of Smac/DIABLO 5 min after treatment, whereas cytochrome c slightly increased at short times. However, cytochrome c levels markedly increased between 2 and 4 h (Fig 1A), which were correlated with increased caspase-3 activity detected by Western blot (Fig 1A) or by enzymatic assay (Fig 1B). Quantitative analysis of Smac/DIABLO and cytochrome c was performed in the blots from three independent experiments clearly showing a differential accumulation of both protein in the cytosol (Fig 1C). This could be due either to differential kinetics of release of both proteins from the mitochondria or to differential stability of these proteins in the cytosol. We confirmed that Smac/DIABLO and cytochrome c were released from the mitochondria with different kinetics by analyzing the levels of both proteins in cytosolic and mitochondrial fractions (Fig 1D). Note that the high levels of Smac/DIABLO and cytochrome c in the mitochondria makes difficult to detect a differential reduction of these proteins in this cellular fraction at short times of osmostress, whereas the differential release is evident when we analyzed the cytosolic fraction (Fig 1D, right panel). Despite all, it is clear that a complete depletion of Smac/DIABLO in the mitochondria occurred at 180 min, whereas of cytochrome c at 240 min (Fig 1D, left panel). We can not discard, however, that an increased stability of Smac/DIABLO in the cytosol compared to cytochrome c could also contribute to the higher levels detected at early times. Importantly, we also detected a rapid activation of calpains at 5–15 min after osmostress, since fodrin α (286 kDa, but with an apparent molecular weight of 240 kDa by Western blot [34]), a known substrate for calpains, was proteolized to the 145–150 kDa forms (cleaved fodrin α) (Fig 1A). Quantitative analysis of this band showed that nearly 20% of fodrin α is proteolyzed at 60 min after osmostress (Fig 1C), when caspase-3 is not active (Fig 1B). A marked increase in the 145–150 kDa was observed at 3 and 4 h, when caspase-3 activity was high, and a third band (120 kDa) appeared as a result of fodrin α proteolysis by caspase-3 [35,36]. Since calpain activity is dependent of Ca^2+^ levels, we analyzed the effect of a calcium chelant. Microinjection of EGTA (0.5 mM, intracellular concentration) clearly reduced the proteolysis of fodrin α induced by osmostress during 2 h, but did not prevent the cleavage of fodrin α at 3 h, when caspase-3 activity was high (Fig 1E). This results clearly demonstrates that initial cleaved of fodrin α induced by osmostress is calpain (and calcium) dependent, whereas late cleavage of fodrin α might be caspase-3 dependent. Accordingly, oocytes incubated with sorbitol for 4 h in the presence of the caspase-3 inhibitor Z-DEVD.fmk showed a marked reduction of cleaved fodrin α, and a total disappearance of the 120 kDa band, specific of caspase-3 proteolysis (Fig 1F). As shown in Fig 1A and 1C hyperosmotic shock also induced a rapid and progressive phophorylation of p38 and JNK, achieving high levels at 1 h and remaining high for 4 h. Next we analyzed the role of calpains, Smac/DIABLO and stress protein kinases in osmostress-induced apoptosis.

### Inhibition of calpains reduces cytochrome c release and caspase-3 activation

**Fig 2:**
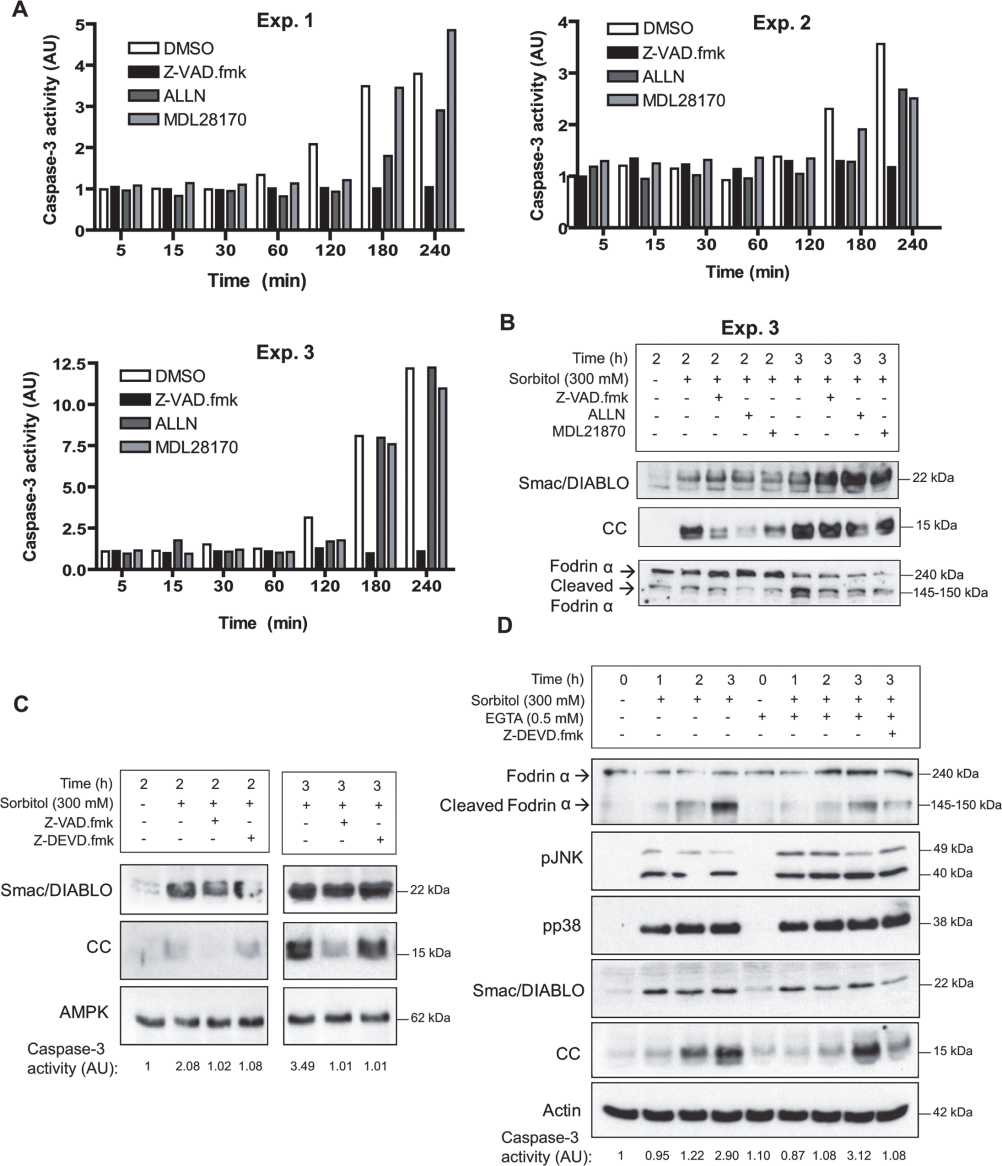
Inhibition of calpains reduces cytochrome c release and caspase-3 activation. A. Calpain inhibitors delay osmostres-induced apoptosis. Oocytes were pre-incubated with calpain inhibitors ALLN or MDL28170, the broad caspase inhibitor Z-VAD.fmk (all at 100 μM), or DMSO (control) for 1 h and treated with 300 mM sorbitol in the presence of inhibitors or DMSO. Caspase-3 activity was determined at different times giving value 1 to non treated oocytes. Data presented from three independent experiments. B. Regulation of cytochrome c release by calpain inhibitors. Smac/DIABLO, cytochrome c (CC), fodrin α, and cleaved fodrin α were determined by Western blot in oocytes treated with 300 mM sorbitol for 2 and 3 h (experiment 3) in the presence or absence of inhibitors. C. Z-VAD.fmk reduces cytochrome c release independently of caspase-3. Oocytes were treated with 300 mM sorbitol for 2 and 3 h in the presence of Z-VAD.fmk (100 μM), caspase-3 inhibitor Z-DEVD.fmk (50 μM), or DMSO (control) and analyzed by Western blot to measure Smac/DIABLO, cytochrome c (CC), and AMPK (loading control). Caspase-3 activity values are indicated below the blots. The result presented is representative of three independent experiments. D. EGTA delays cytochrome c release induced by osmostress. Oocytes were injected with EGTA (0.5 mM final concentration) or with H_2_0 (control) and treated with 300 mM sorbitol for 3 h in the presence or absence of Z-DEVD.fmk (50 μM). Fodrin α, cleaved fodrin α, pJNK, pp38, Smac/DIABLO, cytochrome c (CC), and actin (loading control) were determined by Western blot. Caspase-3 activity is indicated below the blots. The Western blot is representative of three independent experiments.

We measured caspase-3 activity in oocytes treated with 300 mM sorbitol in the presence or absence of calpains inhibitors ALLN, MDL28170, or the general inhibitor of caspases Z-VAD.fmk. Caspase-3 activity was completely blocked by Z-VAD.fmk during the time-course experiment (5 min to 4 h) as expected, and ALLN or MDL28170 clearly inhibited caspase-3 activity between 2 or 3 h, depending of the experiment considered (Fig 2A). However, at 4 h after treatment caspase-3 activity was high in the presence or absence of the inhibitors ALLN or MDL28170. Next, we analyzed by Western blot the release of cytochrome c in a particular experiment (exp. 3). We observed a clear reduction of cytochrome c release at 2 h in the oocytes treated with sorbitol in the presence of ALLN (Fig 2B). MDL28170 and Z-VAD.fmk partially reduced the release of cytochrome c at 2 h. However, 3 h after treatment high levels of cytochrome c release were detected in all conditions assayed (Fig 2B). The effect of Z-VAD.fmk on cytochrome c release was independent of caspase-3, since a specific inhibitor of caspase-3 (Z-DEVD.fmk) did not reduce cytochrome c release at 2 or 3 h after treatment, whereas Z-VAD.fmk did it (Fig 2C). Of note, the calpain inhibitors did not affect the release of Smac/DIABLO (Fig 2B) or the phosphorylation of p38 and JNK (data not shown). The above results suggest that calpains regulate the release of cytochrome c, and therefore EGTA microinjection should reduce osmostress-induced apoptosis. Indeed, as we can see in Fig 2D, EGTA 0.5 mM (final concentration inside the oocyte) delayed the release of cytochrome c induced by osmostress, but did not change Smac/DIABLO release or p38/JNK activation. Interestingly, the combined inhibition of calpains and caspase-3 significantly reduced cytochrome c and Smac/DIABLO release at 3 h (Fig 2D, last lane). This result suggests that positive feedback loops are engaged after caspase-3 activation (see Discussion).

### Blockage of Smac/DIABLO with antibodies inhibits cytochrome c release and caspase-3 activation

**Fig 3:**
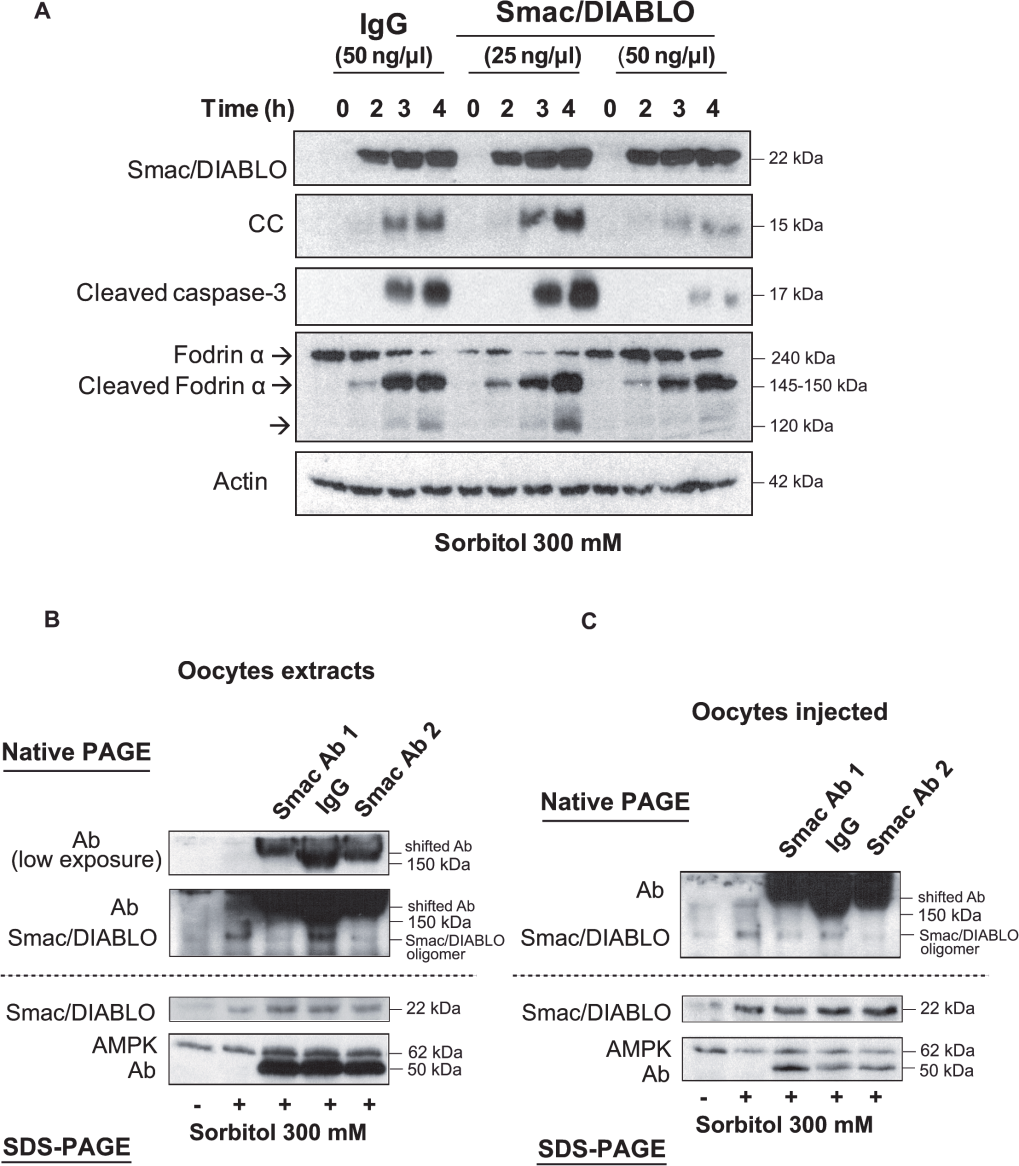
Blockage of Smac/DIABLO with antibodies inhibits cytochrome c release and caspase-3 activation. A. Smac/DIABLO antibodies reduce osmostress induced apoptosis. Oocytes were injected with anti-Smac/DIABLO (25 or 50 ng/μl final concentration) or with anti-human IgG (50 ng/μl) as a control, and treated with 300 mM sorbitol. Cytosolic extracts were obtained at different times to measure Smac/DIABLO, cytochrome c (CC), cleaved caspase-3, fodrin α, cleaved fodrin α, and actin (loading control) by Western blot. The result is representative of three independent experiments. B. Smac/DIABLO antibodies interact with Smac/DIABLO in cytosolic fractions. Oocytes were treated with 300 mM sorbitol and cytosolic extracts were obtained at 3 h and incubated with anti-Smac/DIABLO from ProSci (Ab1), anti-Smac/DIABLO from Calbiochem (Ab2), or rabbit IgG from Sigma as a control antibody. 100 μl of cytosolic extract were incubated with 5 μl of the corresponding antibody (1μg/μl) for 2 h at 4°C on a rotating wheel before their analysis by native-PAGE or SDS-PAGE and Western blot to measure the levels of Smac/DIABLO, AMPK, and the antibodies. C. Smac/DIABLO antibodies interact in vivo with Smac/DIABLO. Oocytes were injected with Ab1, Ab2, or rabbit IgG (50 ng/μl final concentration), and treated with 300 mM sorbitol for 3 h. Cytosolic extracts were analyzed by native-PAGE or SDS-PAGE and Western blot to measure the levels of Smac/DIABLO, AMPK, and the antibodies.

The rapid Smac/DIABLO release from the mitochondria induced by osmostress might be important to regulate cell death in the oocyte. Increased levels of Smac/DIABLO in the cytosol could prime oocytes for apoptosis by inhibition of endogenous inhibitors of caspases (IAPs) before massive cytochrome c release. Since no specific inhibitors of Smac/DIABLO have been reported, we microinjected different amounts of Smac/DIABLO antibodies in *Xenopus* oocytes. 50 ng of anti-Smac/DIABLO significantly reduced cytochrome c release and caspase-3 activation induced by osmostress, compared with a control antibody (Fig 3A); whereas calpain activation, measured as the levels of cleaved fodrin α (150 kDa), was not affected. A clear reduction of the 120 kDa band for fodrin α confirms that caspase-3 was inhibited. These results indicate that early Smac/DIABLO release from the mitochondria regulates late cytochrome c release and caspase-3 activation. To prove that Smac/DIABLO antibodies really interact with Smac/DIABLO in vivo, we performed native PAGE analysis using extracts obtained from oocytes exposed to osmostress and incubated with the antibodies (Fig 3B), or extracts obtained from oocytes injected with the antibodies and exposed to osmostress (Fig 3C), and compared to SDS-PAGE analysis. In both experimental conditions Smac/DIABLO antibodies presented a shift in their electrophoretic mobility and a disappearance of Smac/DIABLO oligomer in native PAGE gels. It has been reported that native Smac/DIABLO behaves as an oligomer in solution with an apparent molecular weight of 100 kDa in a gel filtration column [20]. When we analyzed SDS-PAGE gels Smac/DIABLO monomer (22 kDa) was present in all the samples exposed to osmostress, and the antibodies did not show any shift (Fig 3B and 3C). These data clearly demonstrates that the antibodies interact in vivo with Smac/DIABLO.

### Inhibition of the p38 and JNK signaling pathways by chemical compounds reduces osmostress-induced apoptosis

**Fig 4:**
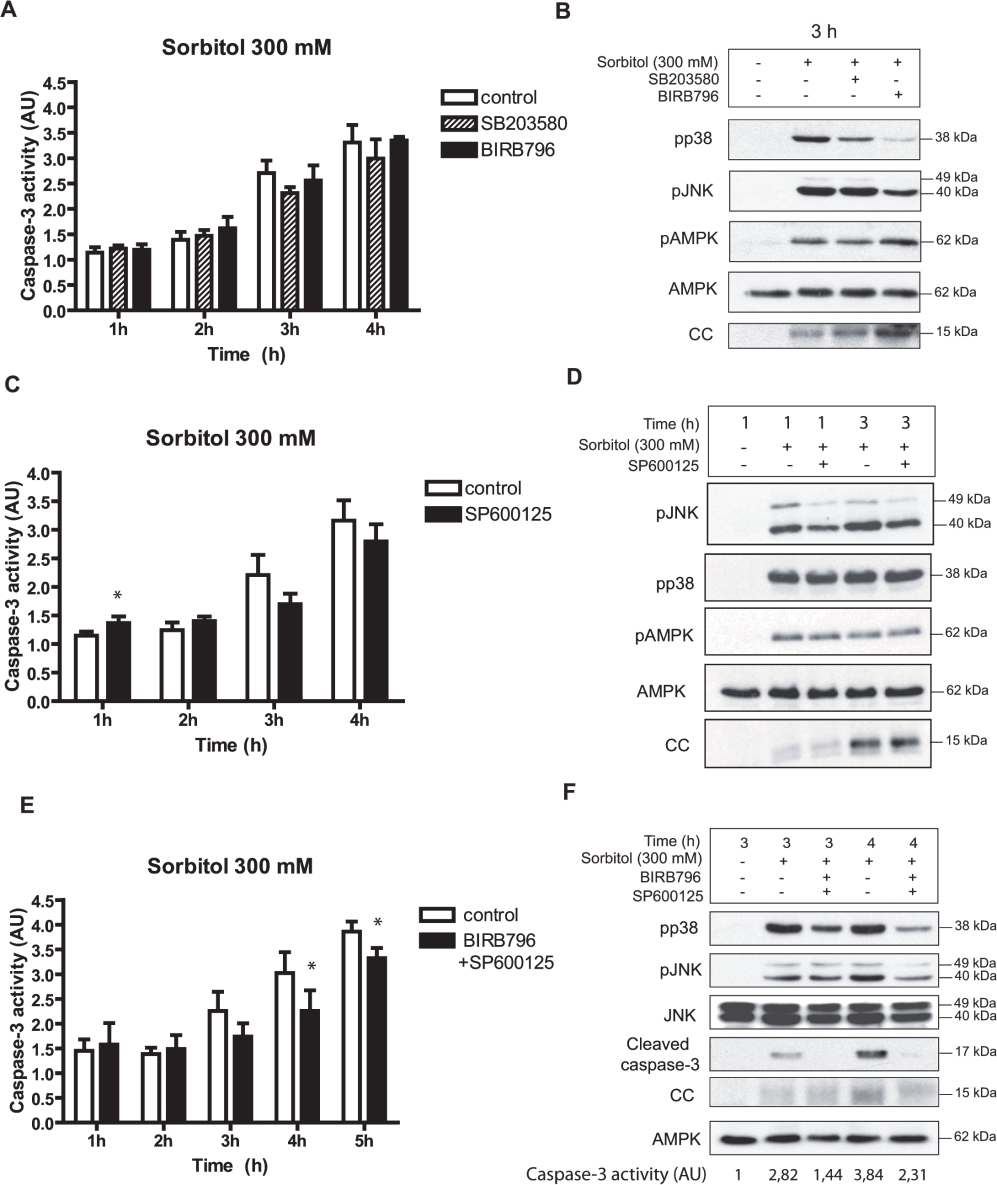
Inhibition of the p38 and JNK signaling pathways by chemical compounds reduces osmostress-induced apoptosis. A and B. p38 inhibitors do not modify osmostress-induced apoptosis. Oocytes were pre-incubated in MBS with SB203580 (100 μM) or BIRB796 (100 μM) for 1 h and then treated with 300 mM sorbitol in the presence of inhibitors. Caspase-3 activity was determined at different times giving value 1 to non treated oocytes. Data are represented as mean ± SEM, (n = 4). pp38, pJNK, pAMPK, AMPK and cytochrome c (CC) release were determined by Western blot at 3 h after treatment. C and D. The JNK inhibitor SP600125 (100 μM) does not reduce osmostress-induced apoptosis. Oocytes were pre-incubated with the inhibitor, treated as described previously, and caspase-3 activity was determined at different times. Data are represented as mean ± SEM (n = 6) and analyzed with Paired t-test comparing control versus inhibitor treatment. * p<0.05. The Western blot shows results at 1 and 3 h after treatment. E and F. BIRB796 + SP600125 treatment reduces osmostress-induced apoptosis. Oocytes were incubated with 100 μM BIRB796 plus 100 μM SP600125, treated as described before, and caspase-3 activity analyzed. Data are represented as mean ± SEM, (n = 3). * p<0.05 (Paired t-test). pp38, pJNK, JNK, cleaved caspase-3, and cytochrome c (CC) release were determined by Western blot at 3 and 4 h after treatment. The Western blots in all figures are representative of at least three independent experiments.

The early activation of p38 and JNK between 15 min and 2 h might have a role in the regulation of hyperosmolar stress-induced apoptosis. We did not observe any significant reduction of cytochrome c release (Fig 4B) and caspase-3 activation (Fig 4A) induced by osmostress in oocytes incubated with the p38 inhibitors SB203580 or BIRB796, which inhibit p38α/β and all the p38 isoforms, respectively. As shown in Fig 4B both compounds did not change AMPK phosphorylation, but BIRB796 partially reduced JNK phosphorylation induced by osmostress, as reported previously [37]. Similarly, the JNK inhibitor SP600125 did not modify caspase-3 activity (Fig 4C) or cytochrome c release induced by osmostres (Fig 4D). The phospho-JNK levels were not altered markedly by SP600125, as reported previously in different cell systems [38,39], and is probably due to the ATP-competitive nature of SP600125, which selectively inhibits kinase activity but not JNK phosphorylation by upstream kinases [40]. Interestingly, we observed a small, but significant, increase in caspase-3 activity 1 h after osmostress in the presence of the JNK inhibitor, which might indicate that JNK has some anti-apoptotic effects at short times of activation (Fig 4C). When *Xenopus* oocytes were incubated with 300 mM sorbitol in the presence of both BIRB796 and SP600125 we observed a significant decrease of caspase-3 activity at 4 h (Fig 4E), which was correlated with a decrease of cleaved caspase-3 measured by Western blot and a reduction in cytochrome c release (Fig 4F). Although statistically significant, caspase-3 inhibition at 5 h was not as great as at 4 h (Fig 4E). The above results suggest that inhibition of both p38 and JNK pathways reduces cytochrome c release and caspase-3 activity, but does not completely block osmostress-induced apoptosis.

### Constitutively active MKK6 or MEKK1 accelerate hyperosmotic shock-induced apoptosis

**Fig 5:**
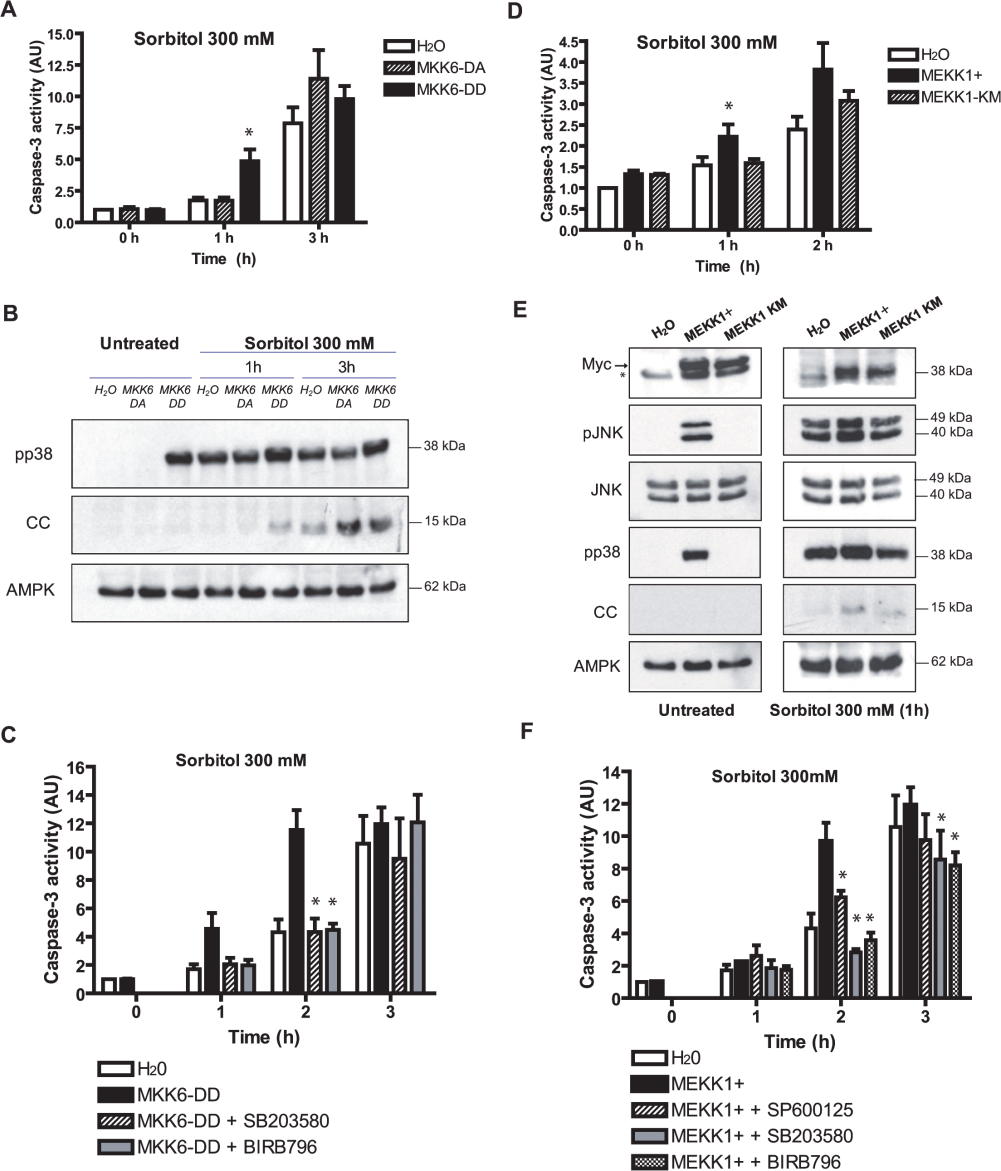
Constitutively active MKK6 or MEKK1 accelerate hyperosmotic shock-induced apoptosis. A and B. MKK6-DD accelerates osmostress-induced apoptosis. Oocytes injected with 50 nl (5ng) of the cRNAs MKK6-DD (constitutively active), MKK6-DA (catalytically inactive), or H_2_O (control) were treated with 300 mM sorbitol for 1 h and 3 h. Caspase-3 activity was determined giving value 1 to water-injected oocytes. Data are represented as mean ± SEM, (n = 4), *p<0.05 comparing with water-injected oocytes (One-way ANOVA and Dunnett Multiple Comparison Test). pp38, cytochrome c (CC), and AMPK (loading control) were analyzed by Western blot. C. MKK6-DD effect on apoptosis is p38-dependent. Oocytes injected with MKK6-DD or H_2_O were pre-incubated in MBS for 18 h in the presence or absence of inhibitors (100 μM) and then incubated with 300 mM sorbitol in the presence or absence of inhibitors. Caspase-3 activity was analyzed as reported previously. Data are represented as mean ± SEM, (n = 3). *p<0.05 comparing with MKK6-DD injected oocytes. D and E. MEKK1+ accelerates osmostress-induced apoptosis. Oocytes were injected with 50 nl (5ng) of the cRNAs MEKK1+ (constitutively active), MEKK1-KM (catalytically inactive), or H_2_O (control) and treated with 300 mM sorbitol. Caspase-3 activity was measured as reported. Data are represented as mean ± SEM, (n = 3), *p<0.05 comparing with water-injected oocytes. pJNK, JNK, pp38, cytochrome c (CC), and AMPK (loading control) were analyzed by Western blot. Expression of the MEKK1+ and MEKK1-KM, containing a myc tag, was confirmed (see arrow). The asterisck (*) indicates an unspecific band detected with the antibody. F. MEKK1+ effect on apoptosis is JNK and p38-dependent. Oocytes treated as in C but with MEKK1+ and the corresponding inhibitors. Data are represented as mean ± SEM, (n = 3), *p<0.05 comparing with MEKK1+ injected oocytes. Western blots in Fig B and E are representative of at least three independent experiments.

To get more insight into the specific role of p38 during hyperosmolar shock-induced apoptosis we expressed a constitutively active MKK6 (DD) or a catalytically inactive MKK6 (DA). Oocytes injected with the different cRNAs and analyzed at 18 h did not show any significant change in caspase-3 activity respect to water-injected oocytes (Fig 5A, see 0 h). Phosphorylation of p38 was elevated in oocytes expressing MKK6-DD, but not in MKK6-DA (Fig 5B). In accordance with caspase-3 activity, untreated oocytes did not present cytochrome c release (Fig 5B). This result clearly shows that prolonged activation of the p38 signaling pathway is not sufficient to induce apoptosis in *Xenopus* oocytes. When oocytes were stressed with sorbitol (300 mM) p38 was activated in all conditions (Fig 5B), but we observed a marked increase in caspase-3 activity 1 h after treatment in the oocytes expressing the constitutively active MKK6-DD (Fig 5A), which was correlated with the release of cytochrome c (Fig 5B). However, oocytes treated for 3 h showed high caspase-3 activity and cytochrome c release in all conditions (Fig 5A and 5B). The acceleration of apoptosis induced by MKK6-DD was blocked by the p38 inhibitors SB203580 or BIRB796 (Fig 5C), suggesting that cytochrome c release and caspase-3 activation could be regulated by the p38α/β isoforms. We also evaluated the role of JNK by expressing a constitutively active MEKK1 (+) or a catalytically inactive MEKK1 (KM). Oocytes expressing MEKK1+ showed increased phosphorylation of JNK, p38 and not significant release of cytochrome c (Fig 5E) or caspase-3 activation (Fig 5D) compared with water or MEKK1-KM-injected oocytes. When oocytes were treated with 300 mM sorbitol, MEKK1+ increased caspase-3 activity at 1 h compared with water or MEKK1-KM-injected oocytes (Fig 5D). Accordingly, cytochrome c release was significantly increased at 1 h in the oocytes expressing MEKK1+ (Fig 5E). The acceleration of caspase-3 activity induced by MEKK1+ was reduced by the inhibitor SP600125, and totally blocked by the inhibitors SB203580 and BIRB796 (Fig 5F), indicating that both JNK and p38 signaling pathways are involved in this process. In conclusion, sustained activation of the p38 pathway by a constitutively active MKK6 or sustained activation of the JNK and p38 pathways by a constitutively active MEKK1+ accelerates hyperosmolar sorbitol-induced apoptosis. These results clearly indicate that both signaling pathways participate in the regulation of osmostress-induced apoptosis.

## Discussion

In this paper we describe different pathways induced by hyperosmotic stress which are integrated in the mitochondria to trigger cytochrome c release and caspase-3 activation. We report for the first time that JNK, p38 and calpain activation in combination with early Smac/DIABLO release from the mitochondria contribute to osmostress-induced apoptosis in *Xenopus* oocytes.

### Calpains regulate osmostress-induced apoptosis

We describe here that hyperosmotic shock induces rapid activation of calpains in *Xenopus* oocytes. Calpain activation might be explained by a transient increase in Ca^2+^ levels, as has been observed in many cell types under hyperosmotic shock [17–19]. Calpains can facilitate apoptosis through the cleavage of various members of the Bcl-2 family [16]. Future studies will address the role of these proteins in osmostress-induced apoptosis. Cleavage of fodrin α, a physiological substrate of calpains, is observed 5 min after osmostress and is increased at 3–4 h, when caspase-3 is activated. It is well know that fodrin α is a substrate of caspase-3 [35,36]; and calpastatin, the natural inhibitor of calpains, is also cleaved by caspase-3 [41,42]. It has been reported that fragmented fodrin lacks the ability to interact with actin [34]. Therefore, calpain activation contributes to cytochrome c release and caspase-3 activity which in turn can increase calpain activation. This positive feedback loop could lead to an irreversible disorganization of the cytosqueleton in the oocyte.

Interestingly, diabetic retinopathy and diabetic cataract are major cause of blindness, and are associated with pathological elevations of Ca^2+^ that leads to the overactivation of calpains [43]. In vivo studies using mouse models indicate that osmotic stress is an important factor contributing to these pathologies [44,45]. Our results suggest that calpain activation induced by hyperosmotic shock could be important for the development of these diseases.

### Smac/DIABLO regulates osmostress-induced apoptosis

This work shows that significant amounts of Smac/DIABLO are released quickly from the mitochondria by hyperosmotic shock, whereas cytochrome c is released at very low amounts and with different kinetics (Fig 1C). The release of Smac/DIABLO became complete and massive at later times (3–4 h), when cytochrome c is also released at higher amounts and caspase-3 is activated. It seems that permeabilization of the outer membrane alone, which would release Smac/DIABLO very fast, is not sufficient to stimulate the release of the same amount of cytochrome c. It has been reported that a disruption of the cytochrome c-cardiolipin interaction seems to be necessary, before or concomitantly with permeabilization of the outer membrane, in order for cytochrome c to be released from the mitochondria [46]. Oxidative modification of cardiolipin, as a consequence of ROS production, might be important for mobilization of the tightly bound pool of cytochrome c [46].

Uren RT el al. have reported that low (non physiological) NaCl concentrations in the lysis buffer (in the range of 10 to 30 mM), or low MgCl_2_ concentrations (in the range of 2 to 4 mM) can avoid the release of cytochrome c in permeabilized mitochondria [47]. These authors found that increasing the salt concentration to 80 mM, or the MgCl_2_ to 8 mM, induced a complete dissociation of cytochrome c. Our lysis buffer contains 250 mM sucrose, 100 mM NaCl, and 2.5 mM MgCl_2_. Since the NaCl concentration used in our experiments is physiological, the differential release of Smac/DIABLO and cytochrome c from the mitochondria is not due to a low salt concentration in the buffer. Moreover, we have supplemented with NaCl (200 mM) or MgCl_2_ (8 mM) our lysis buffer obtaining similar results (data not shown).

Microinyection of antibodies against Smac/DIABLO reduces cytochrome c release from the mitochondria and caspase-3 activation induced by osmostress. How Smac/DIABLO can regulate cytochrome c release? One possibility is that Smac/DIABLO, through inhibition of IAPs, would induce the activation of an unknown caspase that regulates mitochondrial permeabilization. Alternatively, Smac/DIABLO could have another target involved in the control of apoptosis. We have shown that Z-VAD.fmk delays osmostress-induced apoptosis independently of caspase-3. However, it has been reported that Z-VAD.fmk is also a partial inhibitor of calpains and cathepsins [48,49]. Cathepsin inhibitors did not have any effect on cytochrome c release and caspase-3 activity induced by osmostress (data not shown), and Z-VAD.fmk does not inhibit fodrin α proteolysis at 2 h (Fig 2B), indicating that it does not affect early calpain activation. These results suggest that an unknown caspase, activated by Smac/DIABLO, could regulate cytochrome c release.

### p38 and JNK regulate osmostress-induced apoptosis

Although we do not observe a reduction in osmostress-induced apoptosis by using chemical inhibitors separately, the combination of SP600125 and BIRB796 significantly reduces cytochrome c release and caspase-3 activation. Expression of a constitutively active MKK6 or MEKK1 accelerates osmostress-induced apoptosis, indicating that sustained activation of p38 and JNK are pro-apoptotic. In many cell lines, and under certain continuous stimuli, the activation of p38 and JNK is transient, due to down-regulatory mechanisms, including activation of different phosphatases [12,50]. For instance, the activity of MAPKs can be regulated by a family of DUSPs (dual-specificity phosphatases), which are transcriptionally up-regulated by stimuli that activate MAPK signaling, and are thought to play an important role limiting the extent of MAPK activation [51]. In *Xenopus* oocytes the transcriptional effects are not possible and this might explain why p38 and JNK activation in response to osmostress is persistent, in contrast with other cellular systems. Sustained activation of protein kinases favours caspase-3 activation, which in turn might induces constitutive activation of upstream kinases, creating a positive feedback-loop [52].

### Mitochondria integrate stress responses induced by hyperosmotic shock and engage an irreversible death program

**Fig 6:**
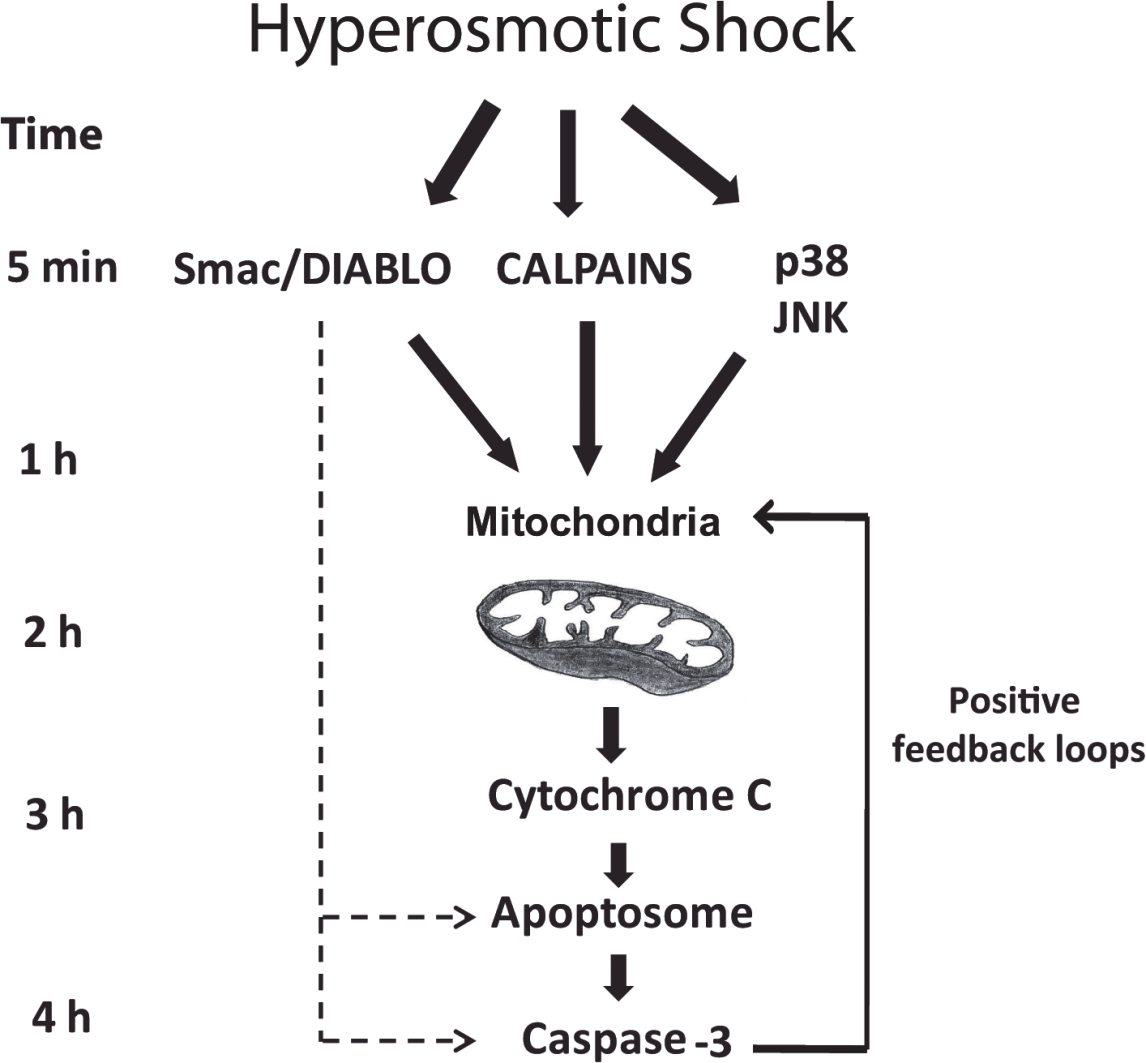
Osmostress-induced apoptosis in *Xenopus* oocytes. Four independent pathways are activated by hyperosmotic shock: JNK, p38, calpains, and Smac/DIABLO. Sustained activation of stress protein kinases in combination with Smac/DIABLO and calpains converge on the mitochondria to induce the release of high levels of cytochrome c into the cytosol, which in turn activates caspase-3 engaging several positive feedback loops to make the cell death program irreversible.

We have shown that hyperosmotic shock induces rapid calpain activation, release of Smac/DIABLO from the mitochondria and JNK/p38 activation. It has physiological sense that a cell under stress must evaluate carefully all the information, since cell death is an irreversible decision. Stress protein kinases (JNK and p38) are perfect sensors to evaluate this situation. They can engage, by phosphorylation of some substrates, a protective response. However, after sustained activation, they can also engage an apoptotic program by phosphorylation of a different set of substrates. Mitochondria would integrate the information received by Smac/DIABLO, calpains and stress protein kinases to make a decision. It is expected to find reversible situations where the cell can recovers if the stress does not persist or gets weaker. A time-course description of the important events induced by hyperosmotic stress that culminates in apoptosis of the oocytes would be as follow (Fig 6): 15–30 min after hyperosmotic stress p38, JNK, and calpains are activated. At the same time Smac/DIABLO is partially released from the mitochondria attaining high levels in the cytosol. At this early stage, there is only a slight release of cytochrome c, which is not sufficient to induce caspase-3 activation. This suggests that opening of the mitochondrial pores might be very fast and transient. 1 h after hyperosmotic shock, p38/JNK protein kinases achieve maximum activity, which can be sustained for several hours. No release of cytochrome c and caspase-3 activation is observed between 1–2 h suggesting that the apoptotic program is still reversible and the cell machinery is evaluating the strength and the duration of the stress, as well as the damage suffered. However, sustained activation of stress protein kinases combined with calpains and Smac/DIABLO will induce the release of significant amounts of cytochrome c, as well as more release of Smac/DIABLO, between 2–4 h after hyperosmotic shock. Now, the high levels of Smac/DIABLO and cytochrome c in the cytosol will induce the formation of the apoptosome and the activation of caspase-3, which in turn would engage different positive feedback loops [41,42,52] making the cell death program irreversible.
